# Effects of Submerged Macrophytes on the Growth, Morphology, Nutritional Value, and Flavor of Cultured Largemouth Bass (*Micropterus salmoides*)

**DOI:** 10.3390/molecules27154927

**Published:** 2022-08-02

**Authors:** Zhaowei Zheng, Zhijuan Nie, Yao Zheng, Xue Tang, Yi Sun, Haojun Zhu, Jiancao Gao, Pao Xu, Gangchun Xu

**Affiliations:** 1 Wuxi Fisheries College, Nanjing Agricultural University, Wuxi 214081, China; zhengzw0427@163.com; 2 Key Laboratory of Integrated Rice-Fish Farming Ecology, Ministry of Agriculture and Rural Affairs, Freshwater Fisheries Research Center (FFRC), Chinese Academy of Fishery Sciences (CAFS), Wuxi 214081, China; zhengy@ffrc.cn (Y.Z.); suny@ffrc.cn (Y.S.); zhuhaojun@ffrc.cn (H.Z.); gaojiancao@ffrc.cn (J.G.); xupao@ffrc.cn (P.X.); 3 State Key Laboratory of Food Science and Technology, Jiangnan University, Wuxi 214122, China; tangxue@jiangnan.edu.cn

**Keywords:** largemouth bass, submerged macrophyte, volatile compounds, nutritional, GC-IMS

## Abstract

Aquaculture environment plays important roles in regulating the growth, morphology, nutrition, and flavor of aquatic products. The present study investigated growth, morphology, nutrition, and flavor formation in largemouth bass (*Micropterus salmoides*) cultured in the ponds with (EM group) and without (M group) the submerged macrophytes (*Elodea nuttallii*). Fish in the EM group showed a significantly greater body length, higher growth rate, and lower hepatosomatic index than those in the M group (*p* *<* 0.05). Moreover, compared with fish in the M group, those in the EM group showed improved muscle quality with significantly elevated levels of crude protein, total free and hydrolysable amino acids, and polyunsaturated fatty acids (*p <* 0.05). Specifically, certain amino acids related to flavor (Glu, Asp, Ala, and Arg) and valuable fatty acids (C18:2, C18:3n3, C20:3n3, and C22:6) were more abundant in the EM group (*p <* 0.05). In addition, the levels of 19 volatile (*p* < 0.05) were significantly higher in the EM group than in the M group. Therefore, *E. nuttallii* significantly improved growth, morphological traits, nutritional components, and characteristic flavor in largemouth bass, indicating the superior nutritional value and palatability of fish cultured with submerged macrophytes.

## 1. Introduction

China is one of the largest producers and consumers of aquatic products in the world, accounting for over 60% of the global aquaculture output [1]. Among aquatic products, the annual production of largemouth bass (*Micropterus salmoides*)—an economically important freshwater aquaculture species [2]—reached about 0.62 million tons [3] with an output value of over $1.76 billion in 2020. This species is native to lakes and rivers in North America [4]. Owing to its rapid growth, delicious flavor, and lack of intermuscular bones, largemouth bass has become widely popular in China since its introduction in 1983 [5]. At present, traditional pond systems remain the only acceptable mode of largemouth bass culture in China [6]. During pond culture, artificial compound feed is supplied, which leads to eutrophication, thereby promoting the outbreak of bloom-forming cyanophytes and diseases and ultimately deteriorating the quality of aquaculture products [7,8]. Therefore, these challenges must be addressed by developing and promoting new ecological aquaculture models.

Ecological aquaculture not only protects the environment but also fulfills the market demand. In recent times, with increased affluence, the focus of consumption has shifted from quantity to quality [9]. Fish meat quality traits, including shape, taste, and flavor, are important factors related to consumer preferences [10], and these attributes are affected by both external and internal drivers, including the culture environment, nutrition, and genetics [11,12,13]. In particular, the nutritional value and sensory traits of fish muscle are affected by the culture environment [14,15]. In bighead carp (*Hypophthalmichthys nobilis*), due to the presence of abundant volatiles with distinct aromas, fish cultured in cold water reservoirs and common culture ponds developed a greater umami intensity than those captured from a natural lake [16].

Therefore, development of effective approaches to produce high-quality aquatic products is a key target in the aquaculture industry [15,17]. In this context, bio-floating beds and submerged plant technology have been used in aquaculture as ecological remediation methods to purify water in situ, thereby improving the meat quality of aquaculture species [15,18]. For instance, floating beds planted with *Ipomoea aquatica* were installed in ponds to assimilate excess nutrients; improve water quality; and promote crab growth, yield, and quality [19]. In grass carp, growth performance and muscle quality improved in the presence of bio-floating beds in culture ponds [15]. In terms of the nutritional quality of aquatic products, the polyculture mode with an in situ ecological floating bed system was proven superior to the conventional monoculture mode [20]. An artificial composite ecosystem with aquatic plants and fish is a reliable eco-agricultural model. To date, however, ecological aquaculture of largemouth bass with aquatic plants has received little attention, and the effects of water quality factors on flavor have seldom been reported.

Volatile compounds significantly affect food flavor, further influencing the overall evaluation of food. Various techniques, including gas chromatography coupled to ion mobility spectrometry (GC-IMS), have been developed to investigate volatile organic compound emissions. In this technique, GC is applied for pre-separation, followed by IMS, and this method does not require time for sample pretreatment [21,22]. Therefore, GC-IMS is a rapid, nondestructive, sensitive, and reliable detection method, and it has gained popularity for exploring trace toxic chemicals and drug residues [23], particularly in the flavor analysis of food and agricultural products [21,24].

However, no study has investigated differences in the morphology and nutritional quality of largemouth bass cultured in the presence or absence of submerged plants (e.g., *Elodea* sp.). Therefore, in the present study, we compared the water quality as well as largemouth bass morphology and nutritional value between two aquaculture modes and constructed a fingerprint of flavor substances using GC-IMS. Further exploration of the key water quality parameters affecting flavor and nutritional value is warranted. Overall, our findings offer novel insights into the effects of submerged plants on the nutritional value and flavor formation of cultured largemouth bass, providing a reference for healthy, sustainable, and ecological culture of this species.

## 2. Materials and Methods

### 2.1. Experimental Design and Sampling

Largemouth bass juveniles of the same age were purchased from Zhanglin Fishery Co., Ltd. (Anhui, China). Six ponds, each covering an area of 0.17 ha, were selected for two treatments with three replicates at the Yang Zhong experimental base: Freshwater Fisheries Research Center (FFRC) and Chinese Academy of Fishery Sciences (CAFS). Three ponds lacked aquatic vegetation (M group), whereas in the remaining three ponds, submerged macrophytes (*Elodea nuttallii*) covered 20% of the superficial area of the bottom (EM group). The experimental period was 90 days (28 June 2021–28 September 2021). At the start of the trial, fish with an initial body weight of 14.50 ± 0.23 g were randomly distributed into six ponds at a stocking density of 43.48 g·m^−3^. The fish were supplied a commercial floating feed containing ≥47% crude protein and ≥5% crude lipid (Xinxin Tianen Aquafeed, Zhejiang, China). Feeding (stopped when >80% largemouth bass were no longer feeding) was performed twice a day at 8–9 a.m. and 5–6 p.m., with 5% feeding ratio.

Samples were collected every month to record growth performance. Body weight, body length, and liver and visceral weights of 30 fish in each group were measured after 24 h of starvation, and the fish anaesthetized with 100 mg·L^−1^ MS-222. At the end of the experimental period, dorsal muscles of 12 fish in each group were sampled, and stored at −80 °C for subsequent analysis. Water samples were collected from the experimental ponds to determine water quality parameters. All animal experiments conformed to the ARRIVE guidelines and were performed following the U.K. Animals (Scientific Procedures) Act, 1986, and the associated guidelines of the EU Directive 2010/63/EU for animal experimentation.

### 2.2. Water Quality Determination

Dissolved oxygen (DO) and pH were measured in situ using a portable multimeter (HQ30D; HACH, Ames, IA, USA) and the YSI Professional Plus system (YSI Inc., Yellow Springs, OH, USA). To test quality, triplicate water samples from each of the treatment sets were transferred to 500 mL polyethylene bottles and the physicochemical parameters of total nitrogen (TN), total phosphorus (TP), and chemical oxygen demand (COD_Mn_) were analyzed as described previously [25]. All samples were filtered using Whatman filter papers with a pore size of 0.45 μm before laboratory analyses.

### 2.3. Biological, Color and Muscle Nutrients Measurements

In the present study, we calculated the weight gain rate (WGR), specific growth rate (SGR), and hepatosomatic index (HSI) as parameters reflecting growth performance, as follows.

WGR (%) = (W_t_ − W_0_) × 100/W_0_

SGR (% day^−1^) = (lnW_t_ − lnW_0_) × 100/days

HSI (%) = (hepatosomatic weight/body weight) × 100

where W_0_ is the initial body weight and W_t_ is the final body weight.

Dorsal and abdominal skin color of largemouth bass cultured under different conditions was measured using a colorimeter (NR10QC Shenzhen Sanen Time Technology Co., Ltd., Shenzhen, China), calibrated with a standard white tile. L*, a*, and b* values were recorded, and the color results were expressed as L* (lightness), a* (−a*: greenness, +a*: redness), and b* (−b*: blueness, +b*: yellowness) [26].

The approximate composition of muscles was investigated following the national standard methods, with three parallel measurements per group. Moisture content was determined according to the AOAC Official Method 930.15 (drying at 105 °C to a constant weight). Ash content was measured according to the AOAC Official Method 942.05 (burning at 550 °C in a muffle furnace) [27]. Crude protein content was determined according to the AOAC Official Method 968.08 (Kjeldahl nitrogen determination method) [28], and crude fat was determined according to the AOAC Official Method 996.06 (Soxhlet extraction method).

For fatty acid analysis, fatty acid methyl esters (FAMEs) were prepared by transesterification with boron trifluoride and methanol, then dissolved in hexane, and the upper organic phase was collected for analysis with an Agilent 7820 A Gas Chromatograph (Agilent Technologies, Inc., Santa Clara, CA, USA) [29].

To determine amino acid content, muscle samples were freeze-dried and ground to powder. Next, 0.1 g samples were accurately weighed and used for amino acid determination. Briefly, the samples were treated with 6 M HCl for acid hydrolysis at 120 °C for 22 h, and then then neutralized with NaOH, and the supernatant was collected for analysis. Free amino acid were adjusted to an appropriate volume with 5% trichloroacetic acid, mixed well, then allowed to stand for 2 h and filtered. Finally, the supernatant was collected for analysis. Amino acid analyses were performed using high-performance liquid chromatography (HPLC) (Ag 1260 HPLC, American Agilent Company), according to the method described by Harimana [5].

### 2.4. Comparison of Fish Muscle Volatile Substances

Volatile compounds were identified using GC-IMS [30]. Muscle samples from each group were weighed and chopped evenly. Each sample was analyzed in triplicate to ensure the reliability of results. Briefly, 3 g samples in 20 mL headspace bottles were randomly selected. The analytical conditions were as follow: headspace incubation = 15 min, temperature = 60 °C, speed = 500 rpm, injection volume = 500 µL, and syringe temperature = 110 °C. GC conditions were as follows: chromatographic column = MXT-5 (15.00 m × 0.53 mm, 1.00 µm i.d.), column temperature = 60 °C, run time = 20 min, and carrier gas = N_2_ (purity ≥ 99.999%). The initial flow rate of the carrier gas was 2 mL·min^−1^ for 2 min, which was increased to 100 mL·min^−1^, and the total run time was 20 min. IMS conditions were as follows: temperature = 45 °C and drift gas flow rate = 150 mL·min^−1^. The retention index (RI) of each compound was calculated. The analytical software supporting the measurement instruments were vocal, three plug-ins (Reporter, Gallery Plot, and Dynamic PCA), and GC-IMS Library Search, which can analyze samples from different perspectives. Spiked and non-spiked samples were measured five times in parallel to calculate the recovery rate and relative standard deviation (RSD).

### 2.5. Statistical Methods and Data Processing

Data collated using Microsoft Excel were expressed as mean ± standard deviation (SD). In SPSS v26.0. (IBM Corporation, Armonk, NY, USA), *t*-test was performed to determine significant differences between the groups. A *p* < 0.05 indicated significant (*), *p* < 0.01 indicated highly significant (**), and *p* < 0.001 indicated extremely highly significant (***) difference.

## 3. Results

### 3.1. Growth Performance and Morphological Characteristics

**Figure 1:**
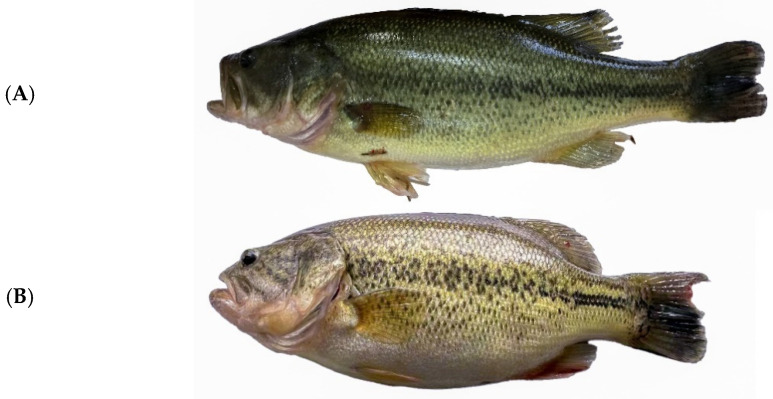
Comparative photographs of *Micropterus salmoides* in the EM (**A**) and M (**B**) groups.

The monthly growth performance of fish is summarized in Table 1. On day 30, there were no significant differences in BL, BT, BW, WGR, or SGR (*p* > 0.05), whereas fish in the EM group showed a significantly lower HSI than those in the M group (*p* < 0.05). On day 60, compared with fish in the M group, those in the EM group showed a significantly higher BW and SGR (*p* < 0.05) and a lower BT and HSI (*p* < 0.05). One month later (at 90 days), the BL, BW, WGR, and SGR of fish in the EM group significantly increased, while HSI continued to decrease significantly (*p* < 0.05). Moreover, fish in the EM group were slender and presented a green body (Figure 1), with darker dorsal skin, as evidenced by significantly lower L* values (Table 2, *p* < 0.01). Regardless of the origin (dorsal or abdominal skin), significant differences were observed in a* and b*.

### 3.2. Water Quality and Dominant Phytoplankton

**Figure 2:**
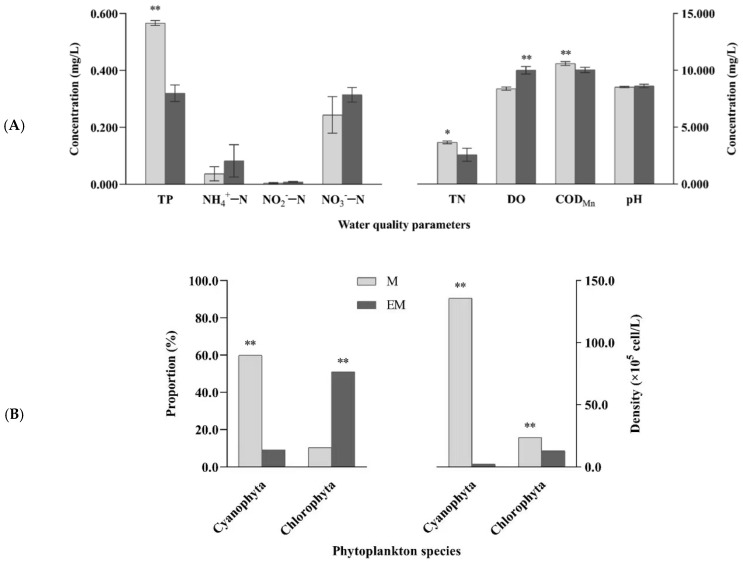
(**A**) Water quality parameters and (**B**) dominant phytoplankton (Cyanophyta and Chlorophyta) in the M and EM group. Note: *p* < 0.05 indicated significant (*), *p* < 0.01 indicated highly significant (**).

Water chemical indices and dominant phytoplankton, including Cyanophyta and Chlorophyta, are shown in Figure 2. At the end of the 90-day experimental period, significant differences in four water quality indices (TN, TP, DO, and COD_Mn_) and dominant phytoplankton reflected the variations in ecological factors for aquaculture water between groups. Compared with values in the M group, TN, TP, and COD_Mn_ in the EM group were significantly decreased, while DO was significantly increased (*p* < 0.05). Furthermore, in the M group, cyanobacteria accounted for 60% of all phytoplankton, with a density of 1.36 × 10^7^ cells·L^−1^, which was significantly higher than that in the EM group (2.28 × 10^5^ cells·L^−1^; *p* < 0.01). More specifically, nearly 60-fold difference was noted between the two groups.

### 3.3. Nutritional Components

The proximate compositions of samples varied (Table 3). All samples were rich sources of proteins. The crude protein content of samples in the M and EM groups was respectively 21.13% and 23.07% (*p* < 0.05). Moisture content was significantly higher in the M group (*p* < 0.01). Ash and crude fat content did not significantly differ between the two groups (*p* > 0.05).

Seventeen free and hydrolysable amino acids were detected in different samples (Table 4). Levels of free amino acids, including Gly, Thr, Tyr, Phe, and Ile, in the muscles of largemouth bass were significantly higher in the EM group than in the M group. Moreover, levels of essential, no−essential, and total free amino acids significantly differed between the two groups (EM > M). Among hydrolysable amino acids, Glu content was the highest in different samples, and Glu content in the EM group was significantly higher than that in the M group. In addition, Asp, Ala, Arg, Ser, His, Thr, Val, Phe, IIe, and Leu levels were higher in the EM group than in the M group.

Furthermore, 22 fatty acids were detected (Table 5), including eight saturated fatty acids (ΣSFAs), five monounsaturated fatty acids (ΣMUFAs), and nine polyunsaturated fatty acids (ΣPUFAs). ΣPUFA levels were higher but ΣSFA and ΣMUFA levels were lower in the EM group than in the M group. In addition, the levels of C18:2, C18:3n3, C20:3n3, and C22:6 (docosahexaenoic acid, DHA), which are important indicators for evaluating the nutritional value of fatty acids, were significantly higher in the EM group. Based on these results, largemouth bass cultured with submerged macrophytes shows a relatively higher nutritional value.

### 3.4. Volatile Compounds

**Figure 3:**
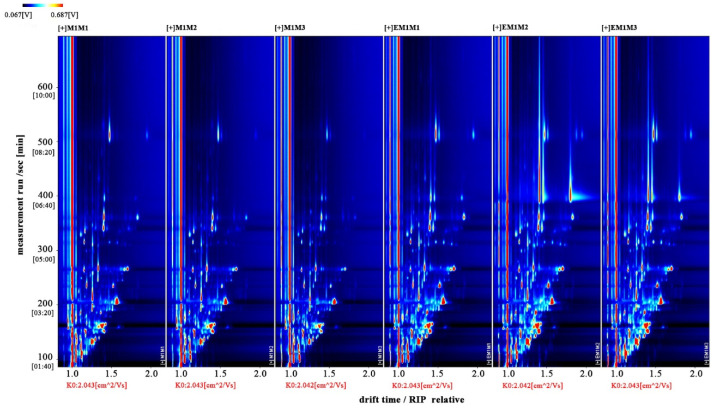
Gas-phase ion mobility spectra of *Micropterus salmoides* muscles. Note: M1M1, M1M2, and M1M3: three samples from the M group. EM1M1, EM1M2, and EM1M3: three samples from the EM group. The background is blue, and the red vertical line on the abscissa (1.0) is the normalized reaction peak. The y-axis represents the retention time of gas chromatography, and the x-axis represents the ion relative drift time. Points on both sides of the reaction peak represent volatile organic compounds. Colors indicate the concentration of substance, with white and red representing a low and high concentration, respectively. The deeper the color, the higher the concentration. For the interpretation of references to colors in this figure legend, please refer the web version of this article.

**Figure 4:**
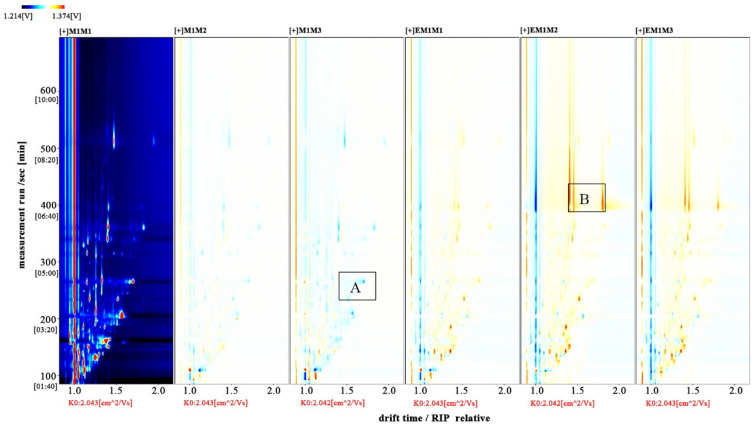
Difference diagram of gas-phase ion mobility spectra of *Micropterus salmoides* muscles. Note: M1M1, M1M2, and M1M3: three samples from the M group. EM1M1, EM1M2, and EM1M3: three samples from the EM group.

**Figure 5:**
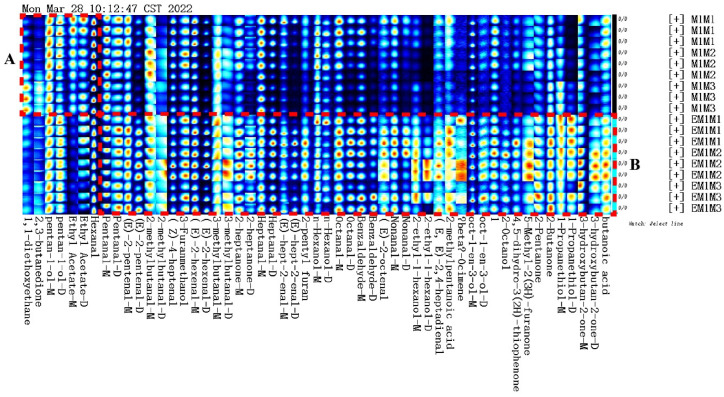
Gallery plot of selected volatile organic compounds in the gas-phase ion migration spectrum. Note: Regions A and B represent the characteristic peak areas of the M and EM group, respectively.

The entire spectrum representing total volatile substances was presented as two-dimensional topographical visualization. Figure 3 shows significant differences in the gas-phase ion migration spectra of muscle samples. The concentration of volatile substances was significantly lower in the M group than in the EM group. We used a different comparison system to visualize the differences between samples. Taking M1 as the reference, the remaining spectral values were deducted from the signal peaks in M1 to obtain the differences in spectra (Figure 4). Substances with levels lower than those in M1 are shown in blue (region A), whereas those with levels higher than those in M1 are shown in red (region B). If the levels of volatile substances are comparable, the background after deduction is white. The deeper the color, the greater the difference. Differences between Figure 3 and Figure 4 clearly demonstrate that the concentration of volatile organic compounds was consistently higher in the EM group. The galleryplot plug-in of the LAV software was used to automatically generate fingerprints of all peaks for determining characteristic differences in volatile substances. As shown in Figure 5, substances related to flavor presented characteristic and common peak areas in the two groups. Regions A and B in Figure 5 represent the characteristic peak areas of the M and EM group, respectively. Therefore, the flavor of samples significantly differed between the M and EM groups.

**Figure 6:**
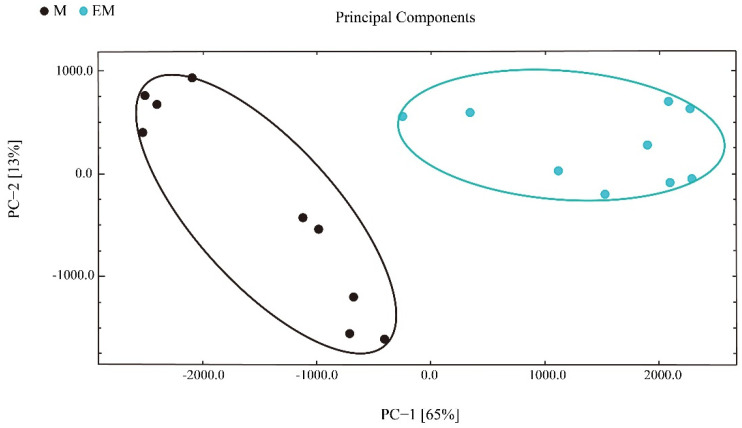
Principal component analysis of flavor compounds in largemouth bass muscle samples.

Next, principal component analysis (PCA) was applied to understand the correlations in largemouth bass muscle samples. PC1 explained 36% sample variance, whereas PC2 explained 28% sample variance (Figure 6). Based on these data, the samples were divided into two groups, and the between-group difference was greater than the within-group difference. Therefore, GC-IMS is suitable to distinguish largemouth bass from different culture models.

In the present study, 55 volatile compounds were identified, of which 54 were qualitative substances, primarily comprising aldehydes, alcohols, ketones, acids, esters, and miscellaneous compounds (Table 6). Twenty-four aldehydes accounted for 43.64% of all volatile compounds and were the most abundant volatile compounds. Thus, different culture environments affect flavor composition. Compared with those in the M group, the levels of aldehydes, namely nonanal-M, nonanal-D, octanal-D, benzaldehyde-M, heptanal-D, 2-methylbutanal-D, and 2-methylbutanal-D, were significantly higher in the EM group. Furthermore, the most abundant alcohols were 2-ethyl-1-hexanol-M, 2-ethyl-1-hexanol-D, 1-propanethiol-D, 1-propanethiol-M, 3-furanmethanol, pentan-1-ol-D, oct-1-en-3-ol-D, and 2-octanol, and their levels were higher in the EM group. Similarly, the levels of ketones, such as 2-butanone, 2-pentanone, 3-hydroxybutan-2-one-D, 3-hydroxybutan-2-one-M, (E)-3-penten-2-one-M, and (E)-3-penten-2-one-D, were significantly higher in the EM group than in the M group. Finally, 2-butanone accounted for approximately 50% of all ketones and was the most abundant ketone in bass muscle in the present study. Thus, 2-butanone appears to be a characteristic volatile compound of largemouth bass.

### 3.5. Correlation Analysis

**Figure 7:**
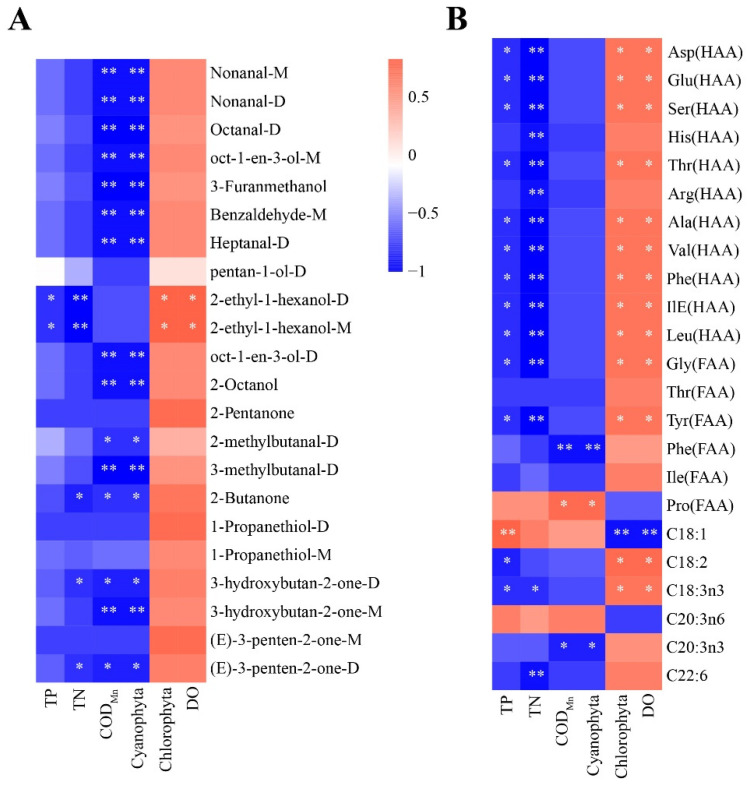
Correlation of significantly different environmental factors with (**A**) volatile compounds and (**B**) nutritional components. Different colors represent the strength of correlation; the red line represents a significant positive correlation, and the blue line represents a significant negative correlation. Note: *p* < 0.05 indicated significant (*), *p* < 0.01 indicated highly significant (**).

To better understand the key factors affecting the flavor and nutritional quality of largemouth bass, we performed Spearman’s correlation analysis. As shown in Figure 7A, the abundance of all volatile substances analyzed was positively correlated with that of DO and chlorophyta, but negatively correlated with that of TN, TP, and COD_Mn_. In addition, 2-ethyl-1-hexanol-D and 2-ethyl-1-hexanol-M were significantly and positively correlated with DO and Chlorophyta but negatively correlated with TN and TP (*p* < 0.05). Cyanobacteria and COD_Mn_ are important biotic and abiotic factors affecting aquatic animals, respectively, and they were significantly but negatively correlated with 68% of the analyzed volatile substances (nonanal-M, nonanal-D, octanal-D, oct-1-en-3-ol-M, 3-furanmethanol, benzaldehyde-M, heptanal-D, oct-1-en-3-ol-D, 2-octanol, 2-methylbutanal-D, 3-methylbutanal-D, 2-Butanone, 3-hydroxybutan-2-one-D,3-hydroxybutan-2-one-M, and (E)-3-penten-2-one-D). Regarding nutritional components (Figure 7B), free and hydrolysable most amino acids were significantly and positively correlated with DO and Chlorophyta and significantly but negatively correlated with TN and TP (*p* < 0.05). ΣPUFAs (C18:2 and C18:3n3) were significantly and positively correlated with DO and Chlorophyta (*p* < 0.05). C20:3n3 levels were significantly but negatively correlated with cyanobacteria and COD_Mn_ (*p* < 0.05), and C22:6 levels were significantly but negatively correlated with TN (*p* < 0.05). Based on these results, cyanobacteria, Chlorophyta, COD_Mn_, DO, TN, and TP may be the key biotic and abiotic factors affecting the flavor and nutritional value of largemouth bass.

## 4. Discussion

### 4.1. Effects of Submerged Macrophytes on the Growth and Morphology of Largemouth Bass

Water quality is a critical factor in aquaculture, as poor-quality water can significantly impede growth and production [31]. In the present study, the higher content of TN, TP, and COD_Mn_ in the M group, which lacked submerged macrophytes, led to cyanobacterial outbreak (1.36 × 10^7^ cells·L^−1^). Conversely, this phenomenon was not observed in the EM group, which comprised submerged macrophytes. TN and TP are the two most important indices of the eutrophication of water bodies [32], while COD_Mn_ is an indicator of organic pollution [33]. Higher values of these indices promote the proliferation of phytoplankton and outbreak of cyanobacterial blooms [34]. Cyanobacteria can produce abundant toxic secondary metabolites, such as dermatoxins, hepatotoxins, and cytotoxins [35], which affect the feeding, growth, and immunity of exposed cultured species [36]. In our experiment, fish in the EM group showed a significantly higher growth rate, corroborating previously reported experimental findings. For instance, Yao showed that the inclusion of live submerged macrophytes in tanks improved the growth of *Macrobrachium nipponense* [37]. Meanwhile, in the present experiment, largemouth bass cultured in the presence of submerged macrophytes were slender, with a green body. Our observations are consistent with reported findings in largemouth bass cultured in an aquaculture system using land-based containers with recycled water [6]. Moreover, fish in the M group showed a significantly higher HSI, suggesting that largemouth bass cultured in the conventional model produced excess body energy, which led to lipid and glycogen accumulation in the liver. Our results are consistent with previous reports from pond and ecological cultures [21]. Overall, aquaculture with submerged macrophytes significantly affected the growth performance and morphology of largemouth bass in the present study, indicating the potential of this model as a reference for farmers.

### 4.2. Effects of sSubmerged Macrophytes on the Nutrient Composition of Largemouth Bass Muscles

Amino acids present a high nutritive value and are important regulators of key metabolic pathways essential for maintenance, growth, feed intake, nutrient utilization, immunity, behavior, and reproduction [38,39]. In the present study, most free and hydrolysable amino acids were more abundant in the EM group; among these, Glu, Asp, Ala, and Arg are well-known as delicious amino acids and contribute significantly to the characteristic flavor of aquatic products [40]. In addition, amino acid content in fish muscles is closely related to their living environment [5,40]. In the present study, levels of 65% amino acids analyzed were significantly and positively correlated with DO and Chlorophyta but significant and negatively correlated with TN and TP (*p* < 0.05). Furthermore, a significant correlation was noted between amino acids and water environmental factors. *Chlorella* is highly effective in counteracting fish enteropathy, maintaining a healthy intestine to balance gene expression [41].

Aquatic products are considered to be nutritionally high-quality foods, because they are rich in amino acids and are an excellent source of unsaturated fatty acids, which are beneficial against cardiovascular disease and promote physiological processes [42,43]. In the present study, the content of C18:2, C18:3n3, C20:3n3, and C22:6 (DHA), which can improve human health and nutritional status [44,45], was significantly higher in the EM group. In particular, DHA is beneficial for optimal brain and neuronal development [46] and is an important indicator for evaluating the nutritional value of fatty acids. Moreover, PUFAs (C18:2 and C18:3n3) were significantly and positively correlated with DO and Chlorophyta (*p* < 0.05). C20:3n3 level was significantly but negatively correlated with cyanobacteria and COD_Mn_ (*p* < 0.05), while C22:6(DHA) level was significantly but negatively correlated with TN (*p* < 0.05). In a previous study on channel catfish, long-term exercise was shown to increase bacterial diversity and richness as well as alter the intestinal microbial composition and unsaturated fatty acid and amino acid biosynthesis [47]. Interestingly, water quality (ammonia) affected swimming activity and feeding behavior [48].

### 4.3. Effects of Submerged Macrophytes on Volatile Compounds in Largemouth Bass Muscles

Each food product has a distinct odor imbued by hundreds of volatile organic compounds, and odor change is one of the most sensitive indicators of food quality. Thus, accurately describing the composition of volatile substances can help assess the quality of agri-food products [23,47]. Various flavor components of largemouth bass meat have been documented [5,6]. In the present study, 24 aldehydes accounted for 43.64% of all components and were the most abundant volatile compounds in largemouth bass muscles. These results confirm that different culture environments indeed affect flavor composition. Aldehydes are mainly generated through lipid oxidation and considered to make the greatest contribution to the flavor of meat products because of their higher content and lower odor detection threshold [49,50].

Compared with values in the M group, the levels of aldehydes, such as nonanal-M, nonanal-D, octanal-D, benzaldehyde-M, heptanal-D, 2-methylbutanal-D, and 2-methylbutanal-D, were significantly higher in the EM group. Such differences in aldehydes and other flavor components have been detected in many aquatic products [51]. Benzaldehyde generates pleasant almondy, fruity, and nutty notes [52] and is an important source of the special aroma of crayfish [53]. Meanwhile, the content of hexanal, which was the most abundant aldehyde, was not significantly different between the two groups. Hexanal has previously been identified as an aroma-active compound providing a green note [54].

Typically, alcohols produce a relatively soft odor, similar to the aroma of fruits [55]. Oct-1-en-3-ol is mainly responsible for the green, plant-like aroma and mushroom-like odor and is formed by the oxidation of arachidonic acid by 12-lipoxygenase [56]. Furthermore, ketones are produced through lipid oxidation and generate creamy and fruity notes [57]. 2-Butanone was abundant in the muscles of largemouth bass in a recirculatory aquaculture system [6]. In the present study, 2-butanone accounted for approximately 50% of all ketones, being the most abundant one. Thus, 2-butanone appears to be a characteristic volatile compound in largemouth bass.

The higher contents of odor compounds in the muscles of fish from the EM group suggest a more pleasant aroma, which may be another reason for their more delicious meat. The differences in volatile substances between the two culture modes can be explained based on two aspects: (1) improvement of water quality by submerged macrophytes through absorption of excess nitrogen and phosphorus, preventing the outbreak of cyanobacteria and (2) abundance of unsaturated fatty acids in fish muscles. Previous studies have shown that cyanobacterial density in ponds and unsaturated fatty acids in fish muscle are linked to the composition of volatile substances [58,59]. In the present study, cyanobacteria and COD_Mn_ were important biotic and abiotic factors affecting fish, respectively, and these factors were significantly but negatively correlated with 68% of the volatile substances analyzed. Thus, our data suggest that submerged plants play an important role in improving the nutritional composition and characteristic flavor profile of *Micropterus salmoides*.

## 5. Conclusions

In summary, largemouth bass cultured in an ecological pond with submerged macrophytes (*Elodea nuttallii*) showed optimal growth, with a slender body shape and significantly higher contents of crude protein, total free and hydrolysable amino acids, and ΣPUFA, compared with fish cultured in conventional ponds. Seven aldehydes, nine alcohols, and six ketones were identified as characteristic volatile components in the muscles of largemouth bass cultured in an ecological pond with submerged macrophytes. Variations in the profiles of volatile components between the two groups are closely linked to the diverse water environments caused by the different aquaculture models. Furthermore, cyanobacteria, Chlorophyta, COD_Mn_, DO, TN, and TP may be the key factors affecting the flavor and nutritional value of largemouth bass. In general, aquaculture with live submerged macrophytes can not only bioremediate the water in situ without producing aquaculture wastewater but also improve the nutritional quality and flavor of aquatic products. Therefore, this is an environmentally friendly and high-value-added ecological aquaculture model worthy of extensive application and popularization.

## Figures and Tables

**Table 1:** Growth performance and morphological indices of largemouth bass.

Item	Time/d	M	EM
BL/cm	30	139.96 ± 8.19	142.69 ± 4.42
	60	191.21 ± 9.00	192.38 ± 11.04
	90	207.13 ± 8.06	221.15 ± 7.67 *
BT/cm	30	21.91 ± 2.01	22.56 ± 1.80
	60	31.22 ± 1.20 *	30.43 ± 1.26
	90	34.51 ± 2.89	36.26 ± 2.00 *
BW/g	30	61.54 ± 8.38	63.32 ± 7.56
	60	163.72 ± 16.71	174.07 ± 18.37 *
	90	214.99 ± 25.37	252.86 ± 22.21 *
WGR/%	30	330.33 ± 58.60	342.77 ± 52.85
	60	168.05 ± 20.72	175.42 ± 9.57
	90	31.23 ± 6.85	45.64 ± 5.36 *
SGR/(%/d)	30	4.83 ± 0.49	4.89 ± 0.40
	60	3.28 ± 0.24	3.38 ± 0.12 *
	90	0.90 ± 0.18	1.25 ± 0.56 *
HSI/%	30	2.82 ± 0.40 *	2.49 ± 0.42
	60	2.23 ± 0.28 *	1.63 ± 0.38
	90	2.09 ± 0.20 *	1.59 ± 0.15

Note: *p* < 0.05 indicated significant (*).

**Table 2:** Chroma values of largemouth bass skin.

Item	Dorsal Skin		Abdominal Skin	
	M	EM	M	EM
L*	49.92 ± 5.81 **	37.78 ± 2.77	68.67 ± 6.11	64.08 ± 3.89
a*	1.07 ± 0.44 ***	−1.52 ± 0.73	2.81 ± 0.91 **	0.93 ± 0.81
b*	14.50 ± 1.13 ***	5.97 ± 1.14	6.25 ± 0.71 ***	2.15 ± 0.47

Note: *p* < 0.01 indicated highly significant (**), and *p* < 0.001 indicated extremely highly significant (***) difference.

**Table 3:** Proximate chemical composition of fish meat (%, *n* = 3).

Items	M	EM	*p*
Moisture	72.63 ± 1.04	69.03 ± 0.40	0.005 **
Ash	1.43 ± 0.06	1.87 ± 0.35	0.103
Crude lipid	3.43 ± 0.71	3.87 ± 0.45	0.422
Crude protein	21.13 ± 0.67	23.07 ± 0.35	0.011 *

Note: *p* < 0.05 indicated significant (*), *p* < 0.01 indicated highly significant (**).

**Table 4:** Amino acid profile (*n* = 3).

	FAA (mg/g)		HAA (g/100 g)	
	M	EM	M	EM
Aspartic acid (Asp)	0.17 ± 0.00	0.16 ± 0.00	4.58 ± 0.24	5.29 ± 0.29 *
Glutamic acid (Glu)	0.45 ± 0.01	0.46 ± 0.00	6.97 ± 0.41	8.08 ± 0.48 *
Serine (Ser)	0.03 ± 0.00	0.03 ± 0.00	1.58 ± 0.09	1.86 ± 0.10 *
Histidine (His)	3.02 ± 0.16	2.96 ± 0.08	1.19 ± 0.05	1.49 ± 0.11 *
Glycine (Gly)	1.69 ± 0.17	2.21 ± 0.07 **	2.26 ± 0.05	2.60 ± 0.21
Threonine (Thr)	0.61 ± 0.02	0.74 ± 0.02 **	1.80 ± 0.08	2.09 ± 0.14 *
Arginine (Arg)	0.04 ± 0.00	0.03 ± 0.01	2.37 ± 0.10	2.73 ± 0.20 *
Alanine (Ala)	1.00 ± 0.05	1.04 ± 0.03	2.57 ± 0.12	2.97 ± 0.18 *
Tyrosine (Tyr)	0.13 ± 0.01	0.15 ± 0.00 *	1.15 ± 0.22	1.35 ± 0.08
Cysteine (Cys-s)	0.04 ± 0.01	0.03 ± 0.00	0.07 ± 0.05	0.09 ± 0.01
Valine (Val)	0.27 ± 0.01	0.27 ± 0.01	2.46 ± 0.16	2.88 ± 0.19 *
Methionine (Met)	0.12 ± 0.01	0.13 ± 0.01	0.94 ± 0.31	1.13 ± 0.05
Phenylalanine (Phe)	0.08 ± 0.00	0.09 ± 0.00 **	1.82 ± 0.11	2.10 ± 0.12 *
Isoleucine (IIe)	0.09 ± 0.00	0.10 ± 0.00 ***	2.12 ± 0.12	2.48 ± 0.15 *
Leucine (Leu)	0.12 ± 0.09	0.18 ± 0.00	3.41 ± 0.21	3.97 ± 0.25 *
Lysine (Lys)	0.52 ± 0.01	0.54 ± 0.01	4.00 ± 0.29	4.69 ± 0.33
Proline (Pro)	0.40 ± 0.05 **	0.23 ± 0.03	1.60 ± 0.18	1.33 ± 0.36
EAA	1.81 ± 0.09	2.05 ± 0.04 *	16.54 ± 1.19	19.33 ± 1.23
NEAA	6.95 ± 0.11	7.30 ± 0.18 *	24.33 ± 1.42	27.79 ± 1.69
TAA	8.76 ± 0.02	9.35 ± 0.20 *	40.87 ± 2.61	47.12 ± 2.89 *

Note: FAA: free amino acids; HAA: hydrolyzed amino acids; EAA: total essential amino acids; NEAA: total no-essential amino acids; TAA: total amino acids. *p* < 0.05 indicated significant (*), *p* < 0.01 indicated highly significant (**), and *p* < 0.001 indicated extremely highly significant (***) difference.

**Table 5:** Fatty acid profile (%, *n* = 3).

Fatty Acid	M	EM
C12:0	0.06 ± 0.01	0.06 ± 0.01
C14:0	3.30 ± 0.08	3.59 ± 0.27
C15:0	0.36 ± 0.02	0.37 ± 0.01
C16:0	22.82 ± 0.20	22.11 ± 0.47
C17:0	0.31 ± 0.01	0.25 ± 0.07
C18:0	3.78 ± 0.15	3.29 ± 0.37
C20:0	0.17 ± 0.01	0.15 ± 0.01
C22:0	0.17 ± 0.08	0.10 ± 0.01
ΣSFA	30.98 ± 0.20 **	29.92 ± 0.30
C14:1	0.07 ± 0.04	0.06 ± 0.02
C16:1	5.71 ± 0.19	5.97 ± 0.29
C17:1	0.39 ± 0.03	0.35 ± 0.01
C18:1	25.24 ± 0.10 **	23.33 ± 0.59
C20:1	0.74 ± 0.08	0.66 ± 0.04
ΣMUFA	32.16 ± 0.25 **	30.37 ± 0.35
C18:2	23.63 ± 0.25	25.64 ± 0.50 **
C18:3n6	0.14 ± 0.02	0.13 ± 0.02
C18:3n3	2.53 ± 0.02	2.64 ± 0.06 *
C20:2	0.48 ± 0.01	0.48 ± 0.00
C20:3n6	0.15 ± 0.01 *	0.13 ± 0.01
C20:3n3	0.17 ± 0.00	0.19 ± 0.01 *
C20:4	0.53 ± 0.03	0.49 ± 0.04
C20:5	1.48 ± 0.15	1.51 ± 0.05
C22:6	7.76 ± 0.19	8.33 ± 0.07 **
ΣPUFA	36.87 ± 0.23	39.54 ± 0.47 **
ΣPUFA/∑SFA	1.19 ± 0.01	1.32 ± 0.02 ***

Note: ΣSFA: total saturated fatty acids; ΣMUFA: total monounsaturated fatty acids; ΣPUFA: total polyunsaturated fatty acids. *p* < 0.05 indicated significant (*), *p* < 0.01 indicated highly significant (**), and *p* < 0.001 indicated extremely highly significant (***) difference.

**Table 6:** Qualitative results of the gas-phase ion mobility spectra of *Micropterus salmoides* muscles (*n* = 3).

Count	Compound	CAS#	Formula	MW	RI	Rt [sec]	Dt [a.u.]	Peak Intensity	
								M	EM
1	Nonanal-M	C124196	C_9_H_18_O	142.2	1112.8	513.223	1.47528	1594.20 ± 569.27	2220.24 ± 352.87 *
2	Nonanal-D	C124196	C_9_H_18_O	142.2	1113.2	513.73	1.95085	222.52 ± 132.59	444.14 ± 156.23 **
3	(E)-2-octenal	C2548870	C_8_H_14_O	126.2	1058.2	434.653	1.33775	230.10 ± 172.74	251.44 ± 100.42
4	2-methylpentanoic acid	C97610	C_6_H_12_O_2_	116.2	1028.9	392.58	1.26318	138.11 ± 95.69	161.91 ± 26.07
5	(E, E)-2,4-heptadienal	C4313035	C_7_H_10_O	110.2	1014.3	371.527	1.19175	195.11 ± 105.12	225.32 ± 80.96
6	Octanal-M	C124130	C_8_H_16_O	128.2	1007.1	361.154	1.40395	1404.23 ± 435.87	1646.58 ± 185.32
7	Octanal-D	C124130	C_8_H_16_O	128.2	1007.3	361.434	1.82706	542.77 ± 398.71	958.61 ± 320.32 *
8	1	unidentified	−	0	990.4	340.757	1.39455	1201.18 ± 324.12	1280.64 ± 185.88
9	oct-1-en-3-ol-M	C3391864	C_8_H_16_O	128.2	984	335.282	1.16248	796.05 ± 170.35	1026.74 ± 115.11 **
10	3-Furanmethanol	C4412913	C_5_H_6_O_2_	98.1	977	329.248	1.10918	332.53 ± 60.43	403.17 ± 56.14 *
11	Benzaldehyde-M	C100527	C_7_H_6_O	106.1	961.3	315.903	1.15223	466.75 ± 161.68	674.45 ± 169.11 *
12	Benzaldehyde-D	C100527	C_7_H_6_O	106.1	960.2	314.96	1.4733	116.41 ± 81.63	322.43 ± 328.21
13	(E)-hept-2-enal-M	C18829555	C_7_H_12_O	112.2	956.1	311.424	1.25978	684.92 ± 388.43	753.34 ± 177.46
14	(E)-hept-2-enal-D	C18829555	C_7_H_12_O	112.2	957.7	312.838	1.67575	204.13 ± 284.33	344.39 ± 361.19
15	Heptanal-M	C111717	C_7_H_14_O	114.2	902.7	265.925	1.33254	1319.31 ± 149.13	1298.56 ± 92.38
16	Heptanal-D	C111717	C_7_H_14_O	114.2	902.2	265.453	1.70106	1117.23 ± 546.80	1592.96 ± 307.47 *
17	(Z)-4-heptenal	C6728310	C_7_H_12_O	112.2	898.9	262.624	1.15065	706.22 ± 133.75	693.12 ± 113.33
18	2-heptanone-D	C110430	C_7_H_14_O	114.2	893.9	258.375	1.63475	127.34 ± 87.70	223.94 ± 109.79
19	n-Hexanol-M	C111273	C_6_H_14_O	102.2	872.7	246.48	1.32617	564.98 ± 71.06	544.49 ± 104.32
20	(E)-2-hexenal-M	C6728263	C_6_H_10_O	98.1	851.9	235.365	1.18317	1160.40 ± 329.61	1239.75 ± 158.51
21	(E)-2-hexenal-D	C6728263	C_6_H_10_O	98.1	847.6	233.025	1.5206	1069.01 ± 1015.98	1662.32 ± 977.41
22	Hexanal	C66251	C_6_H_12_O	100.2	790.8	202.605	1.55949	4991.75 ± 600.01	4903.75 ± 318.76
23	pentan-1-ol-D	C71410	C_5_H_12_O	88.1	770	193.277	1.51613	434.40 ± 117.86	531.57 ± 69.93 *
24	2-ethyl-1-hexanol-D	C104767	C_8_H_18_O	130.2	1032.7	398.037	1.80573	124.18 ± 43.72	846.93 ± 639.43 **
25	2-pentyl furan	C3777693	C_9_H_14_O	138.2	997.1	346.84	1.25753	164.73 ± 67.08	232.93 ± 82.24
26	2-ethyl-1-hexanol-M	C104767	C_8_H_18_O	130.2	1031.2	395.891	1.41016	615.64 ± 225.45	2346.12 ± 1172.02 **
27	β-Ocimene	C13877913	C_10_H_16_	136.2	1046.2	417.35	1.2186	32.92 ± 6.86	51.02 ± 17.46 *
28	pentan-1-ol-M	C71410	C_5_H_12_O	88.1	766.3	191.798	1.25475	653.56 ± 54.47	607.04 ± 67.51
29	oct-1-en-3-ol-D	C3391864	C_8_H_16_O	128.2	983.8	335.066	1.60462	114.15 ± 29.48	185.28 ± 50.23 **
30	n-Hexanol-D	C111273	C_6_H_14_O	102.2	870.6	245.347	1.6415	102.66 ± 26.47	112.21 ± 31.71
31	2-heptanone-M	C110430	C_7_H_14_O	114.2	893.8	258.277	1.26096	376.60 ± 68.28	426.02 ± 44.28
32	2-Octanol	C123966	C_8_H_18_O	130.2	993	342.983	1.80374	66.70 ± 20.87	109.93 ± 34.54 **
33	4,5-dihydro-3(2H)-thiophenone	C1003049	C_4_H_6_OS	102.2	940.8	298.387	1.18131	102.88 ± 47.04	186.07 ± 129.76
34	5-Methyl-2(3H)-furanone	C591128	C_5_H_6_O_2_	98.1	878.9	249.833	1.11789	118.59 ± 40.71	171.50 ± 77.70
35	(E)-2-pentenal-M	C1576870	C_5_H_8_O	84.1	751.9	185.969	1.10931	432.20 ± 106.45	454.48 ± 47.37
36	(E)-2-pentenal-D	C1576870	C_5_H_8_O	84.1	753.4	186.582	1.36359	742.97 ± 779.75	1300.98 ± 769.26
37	Pentanal-M	C110623	C_5_H_10_O	86.1	698.1	164.15	1.18501	371.17 ± 17.61 ***	318.41 ± 31.78
38	Pentanal-D	C110623	C_5_H_10_O	86.1	699.6	164.751	1.42598	484.46 ± 115.69	543.81 ± 64.33
39	2-Pentanone	C107879	C_5_H_10_O	86.1	687.3	159.943	1.37898	630.06 ± 58.16	955.98 ± 114.13 ***
40	2-methylbutanal-M	C96173	C_5_H_10_O	86.1	664.3	153.733	1.16001	130.22 ± 19.39	119.90 ± 13.07
41	2-methylbutanal-D	C96173	C_5_H_10_O	86.1	659.3	152.403	1.39552	84.85 ± 23.11	128.68 ± 36.05 **
42	3-methylbutanal-M	C590863	C_5_H_10_O	86.1	651.6	150.312	1.17203	262.76 ± 35.38	255.62 ± 28.59
43	3-methylbutanal-D	C590863	C_5_H_10_O	86.1	656.2	151.553	1.40848	162.28 ± 33.59	212.25 ± 31.97 **
44	Ethyl Acetate-M	C141786	C_4_H_8_O_2_	88.1	613.8	140.105	1.10042	232.82 ± 56.67 ***	96.49 ± 12.85
45	Ethyl Acetate-D	C141786	C_4_H_8_O_2_	88.1	610.6	139.259	1.3406	242.03 ± 57.78	258.51 ± 72.43
46	2-Butanone	C78933	C_4_H_8_O	72.1	585.5	132.491	1.24941	2444.76 ± 456.05	3285.25 ± 393.55 ***
47	1-Propanethiol-D	C107039	C_3_H_8_S	76.2	626.5	143.526	1.36157	106.78 ± 70.85	500.18 ± 146.62 ***
48	1-Propanethiol-M	C107039	C_3_H_8_S	76.2	620.8	142.008	1.17085	358.20 ± 125.51	550.51 ± 67.43 **
49	3-hydroxybutan-2-one-D	C513860	C_4_H_8_O_2_	88.1	719.1	172.675	1.33338	115.23 ± 30.90	212.55 ± 53.40 ***
50	3-hydroxybutan-2-one-M	C513860	C_4_H_8_O_2_	88.1	720.3	173.16	1.06435	296.69 ± 50.68	405.20 ± 71.00 **
51	(E)-3-penten-2-one-M	C3102338	C_5_H_8_O	84.1	728.7	176.54	1.09091	119.00 ± 24.20	158.03 ± 14.62 ***
52	1,1-diethoxyethane	C105577	C_6_H_14_O_2_	118.2	726.7	175.748	1.13189	68.33 ± 31.71	45.99 ± 9.48
53	(E)-3-penten-2-one-D	C3102338	C_5_H_8_O	84.1	725.1	175.11	1.34845	50.96 ± 19.65	99.06 ± 26.14 ***
54	butanoic acid	C107926	C_4_H_8_O_2_	88.1	816	216.095	1.16109	42.25 ± 6.81	43.85 ± 4.03
55	2,3-butanedione	C431038	C_4_H_6_O_2_	86.1	580.7	131.198	1.17158	103.28 ± 44.52	118.02 ± 18.07

Each row in the figure represents all signal peaks selected in a sample, and each column represents signal peaks of the same volatile compounds in different samples. -M and -D indicate the monomer and dimer of the same substance, and these are presented behind some substance. Numbers indicate unidentified peaks. Note: *p* < 0.05 indicated significant (*), *p* < 0.01 indicated highly significant (**), and *p* < 0.001 indicated extremely highly significant (***) difference.

## Data Availability

Not applicable.
